# Transient Aortitis after COVID-19 mRNA Vaccination

**DOI:** 10.31138/mjr.231020.nht

**Published:** 2023-10-20

**Authors:** Hiroaki Nishioka, Yuuki Okumura

**Affiliations:** Department of General Internal Medicine, Kobe City Medical Centre General Hospital, Kobe City, Hyogo, Japan

**Keywords:** aortitis, COVID-19, mRNA-based vaccine

**Figure 1.**
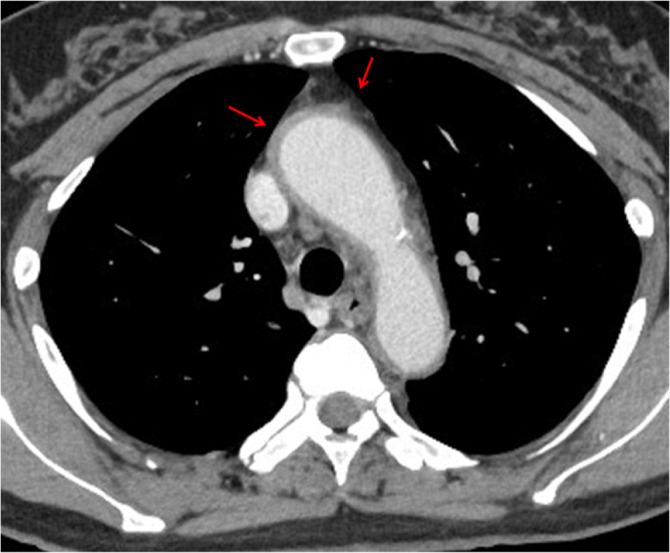
Contrast-enhanced computed tomography reveals thickening of the wall of the ascending aorta and aortic arch with fat stranding (red arrows).

A 62-year-old woman presented with a 5-day history of fever and fatigue that developed the day after receiving the third dose of messenger ribonucleic acid (mRNA)-based vaccine (mRNA-1273, Moderna) for Coronavirus disease 2019 (COVID-19). The patient did not report any other symptoms. Physical examination revealed unremarkable findings, except for a low-grade fever. Laboratory tests showed a white blood cell (WBC) count of 11,300 /μL (neutrophils: 71.8%), C-reactive protein (CRP) level of 16.0 mg/dL, and erythrocyte sedimentation rate of 118 mm/h. The test results for antinuclear antibody, myeloperoxidase-anti-neutrophil cytoplasmic antibodies (ANCA), proteinase-3-ANCA, and rheumatoid factor were negative. Blood culture did not contain any bacteria. Contrast-enhanced computed tomography (CECT) revealed wall thickening of the ascending aorta and aortic arch with fat stranding (**Figure 1**). Ultrasonography did not reveal any abnormal findings in the temporal, subclavian, or carotid arteries. We tentatively diagnosed her with aortitis. We prescribed acetaminophen and followed-up the patient at our outpatient department without any specific intervention. Three days later, her fever relieved, and 4 more days later, her fatigue disappeared, and WBC count and CRP level returned to normal levels. CECT at one month after her first presentation revealed diminishment of wall thickening of the aorta. Her symptoms have not recurred for more than one year. We made a diagnosis of COVID-19 vaccine-associated large-vessel vasculitis in the aorta. The patient’s Naranjo adverse drug reaction probability scale registered at 6 points, which indicated a probable relationship between her symptoms and adverse drug reactions to COVID-19 vaccine.^1^

COVID-19 has spread worldwide and caused almost 15.5 million deaths, which has promoted rapid development of new vaccines. COVID-19 vaccines mainly have two types of formulation: mRNA-based vaccine encoding SARS-CoV-2 spike protein (S) encapsulated in lipid nanoparticles, such as BNT162b2 vaccine (Pfizer/BioNTech) and mRNA-1273 vaccine (Moderna), and adenovirus vector vaccine encoding S protein, such as ChAadOx1 nCoV-19 vaccine (AstraZeneca) and Ad26. COV2.S vaccine (Janssen). These vaccines are effective against COVID-19 and generally safe. However, some immune-mediated adverse events have been reported, such as vasculitis, arthritis, adult-onset Still’s disease, and myocarditis, after COVID-19 vaccination. Vasculitis after COVID-19 vaccination has often manifested as cutaneous small-vessel vasculitis or glomerulonephritis,^2^ while aortitis is rare. Only four cases of aortitis following vaccination have been reported.^3–6^ The patients ranged in age from 52 to 81 years. Three patients had received BNT162b2 vaccine, and one patient had received ChAadOx1 nCoV-19 vaccine. Our patient may be the first to demonstrate aortitis after the administration of Moderna COVID-19 vaccine (mRNA-1273). The differential diagnosis of aortitis includes bacterial aortitis, syphilitic mesoarteritis, Takayasu arteritis, giant cell arteritis, IgG4-related diseases, and Behçet’s disease. Other conditions, including recurrence of malignancy and bacterial infections such as pyelonephritis and spondylitis, can present with similar symptoms to aortitis. Judging from this patient’s clinical course, these diagnoses were less likely in our case. The causal relationship and mechanism between vasculitis and COVID-19 vaccination remain to be elucidated. COVID-19 is known to induce a hyperinflammatory response through activation of innate immune system and overproduction of proinflammatory cytokines.^7^ COVID-19 has been reported to trigger the development and reactivation of autoimmune diseases, including 4 cases of aortitis.^8,9^ Similarly, COVID-19 vaccines is thought to induce an immune response both by direct activation of innate immune system through pattern recognition receptors, and by activating innate and adaptive immune systems by produced S protein,^10^ resulting in an autoinflammatory process.^11^ The optimal treatment for COVID-19 vaccine-associated vasculitis has not yet been established. Some cases reportedly resolve with systemic glucocorticoid therapy;^3,4^ however, some have been successfully treated without steroid therapy.^5,6^ This case suggests that clinicians should consider aortitis in a differential diagnosis in patients suffering from fever without other symptoms after COVID-19 vaccination and should know that such cases can be resolved without immunosuppressive therapy.
